# New records of Sabethini (Diptera: Culicidae) from Colombia

**DOI:** 10.3897/BDJ.10.e68413

**Published:** 2022-02-03

**Authors:** Nelson Naranjo-Díaz, Juan Suaza-Vasco, Jacobo Pineda-Angel, Sandra Uribe

**Affiliations:** 1 Grupo de Investigación en Sistemática Molecular, Facultad de Ciencias, Universidad Nacional de Colombia, Sede Medellín, Calle 59A 63-20. Bloque 16, Laboratorio 102, Medellín, Colombia Grupo de Investigación en Sistemática Molecular, Facultad de Ciencias, Universidad Nacional de Colombia, Sede Medellín, Calle 59A 63-20. Bloque 16, Laboratorio 102 Medellín Colombia

**Keywords:** Sabethini, Colombia, Neotropical Region, records data

## Abstract

**Background:**

In the Neotropical Region, the mosquitoes, grouped in the tribe Sabethini (Arthropoda, Insecta, Diptera: Culicidae) are considered of medical importance by the role that some species may have in arbovirus transmission; also, because they are good bioindicators. More than 400 species are currently recognised and are mainly associated with forest areas. The tribe Sabethini is poorly studied and the information about diversity and distribution for species relating to it is scarce. In Colombia, 54 species of the tribe are known; however, several geographical areas have not been included in the studies for this group and data for recent field collections are not available; therefore, the records are outdated.

**New information:**

This study presents the species list of the Sabethini tribe in Colombia, based on a review of previous publications and recent unpublished data. The list includes 68 species of nine genera and 16 subgenera. The genus *Wyeomyia* has the highest species number (39), followed by *Sabethes* (14). A total of 29 new records are registered and actualized information related to the local distribution in some Departments is presented, including geographic coordinates. In this paper, the distribution records of Sabethini for Colombia are updated, revealing the high diversity of this group in the country and providing some useful information for species that may need surveillance or control.

## Introduction

Tribe Sabethini of the subfamily Culicinae is composed of 432 recognised species of 14 genera (Harbach 2014). In the Neotropical Region, there are reported numerous species in different genera including *Isostomyia* (4 species), *Johnbelkinia* (3), *Limatus* (9), *Onirion* (7), *Runchomyia* (8), *Sabethes* (41), *Shannoniana* (3), *Trichoprosopon* (13) and *Wyeomyia* (139) (Harbach 2014). Sabethini mosquitoes prefer forest environments and exhibit predominantly diurnal feeding behaviour (Lane 1953, Suaza-Vasco et al. 2015). Phytotelmata are known as breeding places for some species, but artificial breeding sites are also used for some of them (Lane 1953, Chaverri et al. 2018).

In the Neotropical Region, the tribe Sabethini is related to arbovirus transmission. The genera *Johbelkinia*, *Limatus*, *Sabethes*, *Trichoprosopon* and *Wyeomyia* include species recognised as potential vectors (De Souza Lopes et al. 1975, Auguste et al. 2010, Bueno-Marí et al. 2015, Navarro et al. 2015, Gravina et al. 2018). Some species, such as *L.durhamii* Theobald, 1901, *L.flavisetosus* de Oliveira Castro, 1935, *Sa.chloropterus* (von Humboldt, 1819) and *T.digitatum* (Rondani, 1848), are recognised vectors of viral encephalitis (Worth et al. 1968, Aitken 1972, Shope et al. 2000, Navarro et al. 2015) . Yellow fever is potentially transmitted by *Sa.cyaneus* (Fabricius, 1805), *Sa.chloropterus*, *Sa.albipivus* Theobald, 1903, *Sa.glaucodaemon* (Dyar and Shannon, 1925) and *Sa.soperi*. Lane & Cerqueira, 1942 (Monath 1988, Navarro et al. 2015). Other species, such as *L.asulleptus* (Theobald, 1903) and *Sa.cyaneus* (Fabricius,1805), are related to Mayaro fever transmission (Muñoz and Navarro 2012, Navarro et al. 2015).

In Colombia, there are no recent studies related to this tribe, except published works by Suaza-Vasco et al. (2015), for coffee growing areas; however, diversity of biomes in the country favours the presence of a high number of species. Here, we present an updated species list of the tribe Sabethini in Colombia, based on historical and our own data.

## Materials and methods

The list of species presented in this study was compiled using the following reports: (Lane and Cerqueira 1942, Stone et al. 1959, Vargas and Díaz Nájera 1959, Barreto and Vernon 1969, Knight and Stone 1977, Heinemann and Belkin 1978, Zavortink 1979a, Zavortink 1979b, Kano 1991, Carrejo and Gonzalez 1992, Olano and Tinke 1993, Zuluaga et al. 1993, Marchon-Silva et al. 1996, Molina et al. 2000, Harbach and Peyton 2000, Barrera et al. 2002, Porter and Wolff E. 2004, Ferro et al. 2008, Parra-Henao and Suárez 2012, Barajas et al. 2013, Rozo-Lopez and Mengual 2015, Suaza-Vasco et al. 2015, Rosero-García et al. 2017, Rosero-García et al. 2018). Unpublished data from a Masters thesis (Cochero Bustamante 2017) and database portals (Gaffigan et al. 2014, SIB 2020) were included, as well as new material collected by the authors records for field-collected material with a buccal aspirator, entomological nets or which was taxonomically identified by the authors were also included, the material being collected by direct sample using a buccal aspirator and entomological net. Additionally, some adults were sampled in Shannon traps located in vegetal covers such as forest, guadual and coffee plantations. The light in the trap was activated during the twilight hours and the attracted adults were collected using a mouth aspirator and entomological net.

Species distribution records were classified into ecoregions (WWF 2015) as follows: Llanos, Apure-Villavicencio Dry Forest, Cordillera Oriental Montane Forest, Magdalena Valley Montane Forest, Magdalena Valley Dry Forest, Cauca Valley Montane Forest, Cauca Valley Dry Forest, North-western Andean Montane Forest, Chocó-Darien Moist Forest, South American Pacific Mangroves, Magdalena-Urabá Moist Forest, Amazon-Orinoco-Southern Caribbean Mangroves, Guajira-Barranquilla Xeric Scrub, Caquetá Moist Forest, Negro-Branco Moist Forest and Catatumbo Moist Forest. The list of species is presented by genus and subgenus; the name of the species includes the authorship and the year of description, followed by notes with references to previous records and finally the review of the historical distribution records, including new records (Department: locality [ecoregion]). The abbreviation “cf.” (meaning "confer" or to be compared with) is used for distinguishing some species names to indicate that most of the diagnostic characters correspond to a given species, but some characters are unclear or not available. “cf.” is a qualifier frequently used in taxonomic records and closely associated with open nomenclature (ON) practice (Sigovini et al. 2016).

## Checklists

### Checklist of the tribe Sabethini from Colombia

#### Isostomyia espini

(Martini, 1914)

B81DCB70-900E-51C4-8F9A-6DBE0BA4D030

##### Distribution

Caquetá: Solano [Caquetá Moist Forests].

##### Notes

Reported by Molina et al. (2000).

#### Johnbelkinia leucopus

(Dyar & Knab, 1906)

55B71BF8-0A79-5BAC-AC4F-D7E3E6785E96

##### Distribution

Antioquia: Hispania [Cauca Valley Montane Forests]. Caquetá: Solano [Caquetá Moist Forests].

##### Notes

Reported by Molina et al. (2000), new record.

#### Johnbelkinia longipes

(Fabricius, 1805)

BC7B769E-26B9-5252-8D36-DAA300FF9A60

##### Distribution

Meta: Restrepo, Villavicencio [Apure-Villavicencio Dry Forests]. Santander: Barrancabermeja, Cimitarra [Magdalena-Urabá Moist Forests]. Valle del Cauca: Buenaventura [Chocó-Darién Moist Forests].

##### Notes

Reported by Barreto-Reyes (1955), Barreto and Vernon (1969), Zavortink (1979a), Barrera et al. (2002), Ferro et al. (2008), SIB (2020).

#### Johnbelkinia ulopus

(Dyar & Knab, 1906)

D3D318D2-4149-5171-AA95-5FD468292D11

##### Distribution

Antioquia: Hispania, Jardín, Valparaíso [Cauca Valley Montane Forests]. Boyacá: Chiquinquirá [Magdalena-Urabá Moist Forests]. Caldas: Anserma, Chinchiná [Cauca Valley Montane Forests]. Cauca: Isla Gorgona [Chocó-Darién Moist Forests]. Chocó: Acandí [Chocó-Darién Moist Forests]. Meta: Restrepo, Villavicencio [Apure-Villavicencio Dry Forests]. Norte de Santander: Villamizar [Catatumbo Moist Forests]. Valle del Cauca: Buenaventura, Darien [Chocó-Darién Moist Forests].

##### Notes

Reported by Knight and Stone (1977), Zavortink (1979b), Suaza-Vasco et al. (2015), SIB (2020), new record.

#### Limatus asulleptus

(Theobald, 1903)

E79C9929-E100-5273-B40E-E557953A08BD

##### Distribution

Caquetá: Solano [Caquetá Moist Forests]. Meta: Villavicencio [Apure-Villavicencio Dry Forests]. Valle del Cauca: Buenaventura [Chocó-Darién Moist Forests].

##### Notes

Reported by Barreto-Reyes (1955), Stone et al. (1959), Heinemann and Belkin (1978), Molina et al. (2000), SIB (2020).

#### Limatus durhamii

Theobald, 1901

CA113FB7-DFC4-5A86-BEB8-DBB4E07DCA92

##### Distribution

Antioquia: Apartadó, Hispania [Cauca Valley Montane Forests]. Caldas: Anserma, Chinchiná [Cauca Valley Montane Forests]. Caquetá: Solano [Caquetá Moist Forests]. Cundinamarca: Guaduas [Magdalena Valley Dry Forests]. Guanía: Inírida [Negro-Branco Moist Forests]. Meta: La Macarena, Puerto López, Puerto Rico, Villavicencio [Apure-Villavicencio Dry Forests, Caquetá Moist Forests, Llanos]. Santander: El Carmen del Chucuri [Magdalena Valley Montane Forests]. Sucre: Coloso [Guajira-Barranquilla Xeric Scrub]. Tolima: Honda, Chaparral [Magdalena Valley Dry Forests].

##### Notes

Reported by Barreto-Reyes (1955), Heinemann and Belkin (1978), Olano and Tinke (1993), Molina et al. (2000), Parra-Henao and Suárez (2012), Barajas et al. (2013), Suaza-Vasco et al. (2015), Cochero Bustamante (2017), SIB (2020).

#### Onirion personatum

(Lutz, 1904)

6797A28F-237B-5FAD-BD4B-F520BF793E57

##### Distribution

Valle del Cauca, Buenaventura [Chocó-Darién Moist Forests].

##### Notes

Reported by Knight and Stone (1977), Harbach and Peyton (2000).

#### Runchomyia (Ctenogoeldia) magna

Theobald, 1905

5E03130F-8FE4-56DA-A760-05A0C1B21063

##### Notes

Reported by Knight and Stone (1977).

#### Sabethes (Peytonulus) identicus

Dyar & Knab, 1907

DBAE62BF-A7C6-5D96-BC75-FFEAFB3DF2DD

##### Distribution

Meta: Puerto López, Villavicencio [Apure-Villavicencio Dry Forests, Cordillera Oriental Montane Forests].

##### Notes

Reported by Gaffigan et al. (2014), SIB (2020).

#### Sabethes (Peytonulus) ignotus

Harbach, 1995

CB2BC5AC-6C8F-5EAC-B9B1-0697AA5112F4

##### Distribution

Caldas: Anserma, Chinchiná [Cauca Valley Montane Forests].

##### Notes

Reported by Harbach (1995), Suaza-Vasco et al. (2015).

#### Sabethes (Peytonulus) luxodens

Hall, Howard & Harbach, 1999

69FF1123-98C1-5B77-BBDB-69168CEA3A2E

##### Distribution

Caldas: Anserma [Cauca Valley Montane Forests].

##### Notes

Reported by Suaza-Vasco et al. (2015).

#### Sabethes (Peytonulus) undosus

(Coquillett, 1906)

4948D68F-80E8-5CD5-8686-ADE2B8C8209F

##### Distribution

Antioquia: Belmira, Jardín, Valparaíso [Cauca Valley Montane Forests]. Caldas: Anserma [Cauca Valley Montane Forests]. Meta: Puerto López [Apure-Villavicencio Dry Forests].

##### Notes

Reported by Heinemann and Belkin (1978), Carrejo and Gonzalez (1992), Barajas et al. (2013), Suaza-Vasco et al. (2015), SIB (2020), new record.

#### Sabethes (Peytonulus) xenismus

Harbach, 1995

EAB8B1F4-EE06-56B1-81E0-320F54FE3071

##### Distribution

Meta: Villavicencio [Apure-Villavicencio Dry Forests].

##### Notes

Reported by Harbach (1995), SIB (2020).

#### Sabethes (Sabethes) albiprivus

Theobald, 1903

4087E7E6-8BF4-5B23-AFE3-43FEB135DB88

##### Distribution

Córdoba: San Bernardo del Viento [Magdalena-Urabá Moist Forests].

##### Notes

Reported by Barreto-Reyes (1955), new record.

#### Sabethes (Sabethes) belisarioi

Neiva, 1908

6C8872F7-7F8A-5D54-B51E-AF82F1F737FB

##### Distribution

Caquetá: Solano [Caquetá Moist Forests]. Valle del Cauca: Buenaventura [Chocó-Darién Moist Forests].

##### Notes

Reported by Barreto-Reyes (1955), Stone et al. (1959), Vargas and Díaz Nájera (1959), Barreto and Vernon (1969), Molina et al. (2000).

#### Sabethes (Sabethes) cyaneus

(Fabricius, 1805)

B7E6DEB6-C862-5A8D-A884-8E2DBD24CDEC

##### Distribution

Caquetá, Solano [Caquetá Moist Forests]. Córdoba: San Bernardo del Viento [Magdalena-Urabá Moist Forests]. Meta: Restrepo [Apure-Villavicencio Dry Forests]. Sucre: Coloso [Guajira-Barranquilla Xeric Scrub]. Valle del Cauca: Buenaventura [Chocó-Darién Moist Forests].

##### Notes

Reported by Barreto-Reyes (1955), Stone et al. (1959), Barreto and Vernon (1969), Heinemann and Belkin (1978), Molina et al. (2000), Cochero Bustamante (2017), SIB (2020).

#### Sabethes (Sabethes) quasicyaneus

Peryassú, 1922

DBE754C9-99F1-5116-A281-C554A95D3CD0

##### Distribution

Santander: San Vicente de Chucurí [Magdalena Valley Montane Forests].

##### Notes

Reported by Barreto-Reyes (1955), Stone et al. (1959), Knight and Stone (1977).

#### Sabethes (Sabethes) tarsopus

Dyar & Knab, 1908

C40A9EB2-F875-5148-B050-96E5FA5A6A84

##### Notes

Reported by Barreto-Reyes (1955).

#### Sabethes (Sabethinus) intermedius

(Lutz, 1904)

5802977A-9AB4-5D74-A45A-CEC09DD6B57B

##### Distribution

Caquetá: Solano [Caquetá Moist Forests]. Chocó: Nuquí [Chocó-Darién Moist Forests]. Risaralda: San Julian [Cauca Valley Montane Forests]. Valle del Cauca: Buenaventura [Chocó-Darién Moist Forests].

##### Notes

Reported by Barreto-Reyes (1955), Stone et al. (1959), Barreto and Vernon (1969), Harbach (1994), Molina et al. (2000), Suaza-Vasco et al. (2015), new record.

#### Sabethes (Sabethinus) cf. xhyphydes

Harbach, 1994

10195BC7-80DB-5D0A-A5E3-4A40E912FE9B

##### Distribution

Caldas: Anserma [Cauca Valley Montane Forests].

##### Notes

New record

#### Sabethes (Sabethoides) chloropterus

(von Humboldt, 1819)

3B21D5B7-DA51-57D9-A5C2-87CD19065D90

##### Distribution

Antioquia: Hispania [Cauca Valley Montane Forests]. Caquetá: Solano [Caquetá Moist Forests]. Caquetá: Solano [Caquetá Moist Forests]. Meta: Villavicencio [Apure-Villavicencio Dry Forests]. Valle del Cauca: Buenaventura [Chocó-Darién Moist Forests].

##### Notes

Reported by Barreto-Reyes (1955), Stone et al. (1959), Barreto and Vernon (1969), Molina et al. (2000), Suaza-Vasco et al. (2015), SIB (2020).

#### Sabethes (Sabethoides) glaucodaemon

(Dyar and Shannon, 1925)

61176A1F-11D4-5E49-96C1-13B6C05CE715

##### Distribution

Antioquia: Valparaíso [Cauca Valley Montane Forests]. Caquetá: Solano [Caquetá Moist Forests].

##### Notes

Reported by Molina et al. (2000), new record.

#### Shannoniana fluviatilis

(Theobald, 1903)

DF0C1BF6-E74A-5A86-85ED-C18465556BC4

##### Distribution

Antioquia: Jardín [Cauca Valley Montane Forests]. Valle del Cauca: Buenaventura [Chocó-Darién Moist Forests].

##### Notes

Reported by Barreto-Reyes (1955), Barreto and Vernon (1969), Suaza-Vasco et al. (2015).

#### Trichoprosopon andinum

Levi-Castillo, 1953

30A9B986-3574-5037-BD52-B1BF6A22D342

##### Distribution

Antioquia: Jericó [Cauca Valley Montane Forests]. Valle del Cauca: Cali [Cauca Valley Dry Forests].

##### Notes

Reported by Carrejo and Gonzalez (1992), Rosero-García et al. (2018), SIB (2020).

#### Trichoprosopon compressum

Lutz, 1905

B7D8BBC1-9305-50B3-9460-28722F25ED70

##### Distribution

Antioquia: Apartadó, La Pintada [Cauca Valley Montane Forests]. Caldas: Anserma, Chinchiná [Cauca Valley Montane Forests]. Meta: Restrepo. [Apure-Villavicencio Dry Forests].

##### Notes

Reported by Lane and Cerqueira (1942), Barreto-Reyes (1955), Parra-Henao and Suárez (2012), Suaza-Vasco et al. (2015), new record.

#### Trichoprosopon digitatum

(Rondani, 1848)

C301EB7F-E665-5084-807B-570EF90C1DD1

##### Distribution

Antioquia: Apartado, Carepa, Hispania, Maceo, La Pintada, Puerto Berrio [Chocó-Darién Moist Forests, Magdalena Valley Montane Forests, Cauca Valley Dry Forests]. Caldas: Anserma, Chinchiná [Cauca Valley Montane Forests]. Caquetá: Solano [Caquetá Moist Forests]. Cundinamarca: Soacha [Magdalena Valley Montane Forests]. Meta: Villavicencio [Apure-Villavicencio Dry Forests]. Quindío: Salento [Cauca Valley Montane Forests]. Santander: Carmen del Chucurí [Magdalena Valley Montane Forests]. Valle del Cauca: Alcalá, Buenaventura [Cauca Valley Montane Forests, Chocó-Darién Moist Forests].

##### Notes

Reported by Vargas and Díaz Nájera (1959), Barreto-Reyes (1955), Barreto and Vernon (1969), Molina et al. (2000), Parra-Henao and Suárez (2012), Barajas et al. (2013), Suaza-Vasco et al. (2015), SIB (2020), new record.

#### Trichoprosopon evansae

Antunes, 1942

FF66F074-5F76-58C1-8BFF-B89100703D1B

##### Distribution

Antioquia: Jardín [Cauca Valley Montane Forests]. Caldas: Rio Sucio [Cauca Valley Montane Forests]. Meta: Restrepo, Villavicencio [Apure-Villavicencio Dry Forests]. Valle del Cauca: Buenaventura [Chocó-Darién Moist Forests].

##### Notes

Reported by Lane and Cerqueira (1942), Barreto-Reyes (1955), Barreto and Vernon (1969), Marchon-Silva et al. (1996), Rozo-Lopez and Mengual (2015), Suaza-Vasco et al. (2015).

#### Trichoprosopon lanei

(Antunes, 1937)

16C77CFC-9BB7-57CC-A45E-CF462EBA86B7

##### Distribution

Meta: Restrepo [Apure-Villavicencio Dry Forests].

##### Notes

Reported by Barreto-Reyes (1955), Stone et al. (1959), Marchon-Silva et al. (1996).

#### Trichoprosopon pallidiventer

(Lutz, 1905)

D79F5039-FD6C-5AC9-A8D6-449AAA444D5D

##### Distribution

Tolima: Guamo [Magdalena Valley Dry Forests]. Valle del Cauca: Alcalá, Buenaventura [Cauca Valley Montane Forests, Chocó-Darién Moist Forests]. Cauca: Puerto Tejada [Cauca Valley Dry Forests].

##### Notes

Reported by Barreto-Reyes (1955), Stone et al. (1959), Barreto and Vernon (1969), Heinemann and Belkin (1978), Suaza-Vasco et al. (2015), SIB (2020), new record.

#### Wyeomyia (subgenus uncertain) cf. argenteorostris

(Bonne-Wepster & Bonne, 1920)

F51B6553-5806-5FCA-983F-72D264FC0754

##### Distribution

Caldas: Anserma [Cauca Valley Montane Forests]. Chocó: Acandí, Litoral de San Juan, Nuquí [Chocó-Darién Moist Forests, South American Pacific Mangroves, Amazon-Orinoco-Southern Caribbean Mangroves].

##### Notes

New record.

#### Wyeomyia (subgenus uncertain) chalcocephala

Dyar & Knab, 1906

D497D2B1-822B-58FA-9F84-0A408B513D54

##### Distribution

Valle del Cauca: Buenaventura [Chocó-Darién Moist Forests].

##### Notes

Reported by Knight and Stone (1977), SIB (2020).

#### Wyeomyia (subgenus uncertain) clasoleuca

Dyar & Knab, 1908

D4F15B02-4F25-575F-A2F5-80F728CEFA48

##### Distribution

Antioquia: Hispania [Cauca Valley Montane Forests]. Caldas: Chinchiná [Cauca Valley Montane Forests]. Valle del Cauca: Buenaventura [Chocó-Darién Moist Forests].

##### Notes

Reported by Barreto-Reyes (1955), SIB (2020), new record.

#### Wyeomyia (subgenus uncertain) melanocephala

Dyar & Knab, 1906

F5921B62-4A97-5914-AED4-7F63F3A2B8B4

##### Distribution

Valle del Cauca: Buenaventura [Chocó-Darién Moist Forests].

##### Notes

Reported by Barreto-Reyes (1955), Stone et al. (1959), Barreto and Vernon (1969).

#### Wyeomyia (subgenus uncertain) moerbista

(Dyar & Knab, 1919)

50BDCAC8-0425-5197-AFD3-9C0DE2FDFCFF

##### Distribution

Antioquia: Apartadó [Magdalena-Urabá Moist Forests].

##### Notes

Reported by Barreto-Reyes (1955), Parra-Henao and Suárez (2012).

#### Wyeomyia (subgenus uncertain) phroso

Howard, Dyar & Knab, 1915

69253888-25F1-5C18-9316-3E4FE3B58980

##### Distribution

Antioquia: Jardín [Cauca Valley Montane Forests]. Caldas: Anserma, Chinchiná [Cauca Valley Montane Forests].

##### Notes

Reported by Suaza-Vasco et al. (2015), Rosero-García et al. (2017), new record.

#### Wyeomyia (subgenus uncertain) serratoria

(Dyar & Nunez Tovar, 1927)

709AF8FA-7AB1-5070-85C0-3E3FFA259058

##### Notes

Reported by Barreto-Reyes (1955).

#### Wyeomyia (subgenus uncertain) undulata

del Ponte & Cerqueira, 1938

4D2FC4E7-4F46-544D-A06B-874801A6B82B

##### Distribution

Antioquia: Hispania [Cauca Valley Montane Forests]. Caldas: Anserma, Chinchiná [Cauca Valley Montane Forests]. Quindío: Quimbaya [Cauca Valley Montane Forests].

##### Notes

Reported by Suaza-Vasco et al. (2015), new record.

#### Wyeomyia (Antunesmyia) colombiana

Lane, 1945

466861EB-7912-5E03-91FC-E0293BCBC2D4

##### Distribution

Meta: Restrepo [Apure-Villavicencio Dry Forests].

##### Notes

Reported by Barreto-Reyes (1955), Stone et al. (1959), SIB (2020).

#### Wyeomyia (Antunesmyia) flavifacies

Edwards, 1922

F8919836-D445-53C4-A6C2-E7D53B0ABD9B

##### Distribution

Antioquia: Apartadó, Turbo [Magdalena-Urabá Moist Forests].

##### Notes

Reported by Barreto-Reyes (1955), Stone et al. (1959), Parra-Henao and Suárez (2012).

#### Wyeomyia (Cruzmyia) kummi

Lane & Cerqueira, 1942

0A2583D9-C922-5A92-8EEC-AD88A3515DB9

##### Notes

Reported by Knight and Stone (1977).

#### Wyeomyia (Cruzmyia) mattinglyi

Lane, 1953

BCDD2393-5E86-5FEC-A1AF-85B93819EE85

##### Notes

Reported by Knight and Stone (1977).

#### Wyeomyia (Decamyia) cf. felicia

(Dyar & Nunez Tovar, 1927)

9C25AD91-E51E-59BB-A772-584CF35E674B

##### Distribution

Chocó: Nuquí [Chocó-Darién Moist Forests].

##### Notes

New record.

#### Wyeomyia (Decamyia) pseudopecten

Dyar & Knab, 1906

ED5FD544-11A4-50A3-B222-B7DA92AB6AFB

##### Distribution

Antioquia: Abejorral [Cauca Valley Montane Forests]. Valle del Cauca: Buenaventura [Chocó-Darién Moist Forests].

##### Notes

Reported by Barreto-Reyes (1955), Heinemann and Belkin (1978), Suaza-Vasco et al. (2015), SIB (2020).

#### Wyeomyia (Decamyia) ulocoma

(Theobald, 1903)

10814275-E837-508E-B985-7E0A7C4289FC

##### Distribution

Antioquia: Fredonia [Cauca Valley Montane Forests]. Caldas: Chinchiná [Cauca Valley Montane Forests]. Quindío: Quimbaya [Cauca Valley Montane Forests]. Valle del Cauca: Buenaventura [Chocó-Darién Moist Forests].

##### Notes

Reported by Carrejo and Gonzalez (1992), Heinemann and Belkin (1978), Suaza-Vasco et al. (2015), SIB (2020).

#### Wyeomyia (Dendromyia) complosa

(Dyar, 1928)

321F2B78-5E2E-564D-A91C-795A708BE22D

##### Distribution

Valle del Cauca: Buenaventura [Chocó-Darién Moist Forests].

##### Notes

Reported by Barreto-Reyes (1955), Stone et al. (1959), Barreto and Vernon (1969).

#### Wyeomyia (Dendromyia) jocosa

(Dyar & Knab, 1908)

8CD67940-3098-5187-8AC7-FBB96F600B92

##### Distribution

Valle del Cauca: Buenaventura [Chocó-Darién Moist Forests].

##### Notes

Reported by Barreto and Vernon (1969), Knight and Stone (1977).

#### Wyeomyia (Dendromyia) luteoventralis

Theobald, 1901

2484F279-9D4A-5C88-9F07-04CCEF3FED02

##### Distribution

Antioquia: La Pintada [Cauca Valley Montane Forests].

##### Notes

Reported by Rozo-Lopez and Mengual (2015).

#### Wyeomyia (Dendromyia) ypsipola

Dyar, 1922

3A31D1C4-834B-5F2A-8771-ADCE007C2E85

##### Distribution

Meta: Villavicencio [Apure-Villavicencio Dry Forests].

##### Notes

Reported by Barreto-Reyes (1955), SIB (2020).

#### Wyeomyia (Dodecamyia) aphobema

Dyar, 1919

E5D2FB88-0126-5A1B-BEAC-40CCDD42F204

##### Distribution

Chocó: Nuquí [Chocó-Darién Moist Forests]. Meta: Restrepo, Villavicencio [Apure-Villavicencio Dry Forests].

##### Notes

Reported by Barreto-Reyes (1955), Stone et al. (1959), Heinemann and Belkin (1978), SIB (2020), new record.

#### Wyeomyia (Exallomyia) tarsata

Lane & Cerqueira, 1942

AA8D8A7F-EC11-55C4-B1BF-59AC5828DA43

##### Distribution

Valle del Cauca: Buenaventura [Chocó-Darién Moist Forests].

##### Notes

Reported by Barreto and Vernon (1969), Knight and Stone (1977).

#### Wyeomyia (Hystatomyia) chocoensis

Porter & Wolff, 2004

EA49171D-05FB-5D76-B46A-6C306DFC5FD5

##### Distribution

Choco: Bahía Solano, Nuquí [Chocó-Darién Moist Forests].

##### Notes

Reported by Porter and Wolff E. (2004), new record.

#### Wyeomyia (Hystatomyia) cf. circumcincta

Dyar & Knab, 1907

D0B2F2EB-250E-5D81-97A5-A268FFDECB02

##### Distribution

Antioquia: Carepa [Chocó-Darién Moist Forests]. Caldas: Anserma, Chinchiná [Cauca Valley Montane Forests]. Choco: Acandí, Litoral del San Juan, Nuquí [Chocó-Darién Moist Forests, South American Pacific Mangroves, Amazon-Orinoco-Southern Caribbean Mangroves].

##### Notes

New record.

#### Wyeomyia (Hystatomyia) intonca

Dyar & Knab, 1910

D5A218E4-A2D6-55DE-918E-EA6584B44C76

##### Distribution

Choco: Quibdo [Chocó-Darién Moist Forests].

##### Notes

Reported by Porter and Wolff E. (2004).

#### Wyeomyia (Miamyia) codiocampa

Dyar & Knab, 1907

8A0CDAAB-3121-5BC1-BCA1-D2007BFC7BCC

##### Distribution

Meta: Puerto López, Villavicencio [Apure-Villavicencio Dry Forests, Llanos].

##### Notes

Reported by Carrejo and Gonzalez (1992), SIB (2020).

#### Wyeomyia (Miamyia) hosautos

Dyar & Knab, 1907

26FB2F29-80B7-51D0-933A-2C52E9EA19B7

##### Distribution

Valle del Cauca: Buenaventura [Chocó-Darién Moist Forests].

##### Notes

Reported by Barreto-Reyes (1955), Stone et al. (1959), Barreto and Vernon (1969).

#### Wyeomyia (Miamyia) cf. limai

Lane & Cerqueira, 1942

625BD6CB-E951-5619-8207-5755CCE04A31

##### Distribution

Antioquia: Jericó [Cauca Valley Montane Forests]. Choco: Litoral del San Juan [South American Pacific Mangroves].

##### Notes

New record.

#### Wyeomyia (Miamyia) oblita

(Lutz, 1904)

9CE5A43E-543B-516C-9215-1D4091B1BE05

##### Distribution

Antioquia: Betania, Hispania [Cauca Valley Montane Forests]. Caldas: Anserma, Chinchiná [Cauca Valley Montane Forests]. Choco: Acandí [Chocó-Darién Moist Forests]. Magdalena: Santa Marta [Guajira-Barranquilla Xeric Scrub].

##### Notes

Reported by Barajas et al. (2013), Suaza-Vasco et al. (2015), new record.

#### Wyeomyia (Nunezia) bicornis

(Root, 1928)

D8C8B7D9-3144-5DC3-90C6-9165B561D391

##### Distribution

Antioquia: Carepa, Ciudad Bolívar, Jardín, Jericó, Támesis, Tarso [Cauca Valley Montane Forests, Chocó-Darién Moist Forests]. Caldas: Anserma, Chinchiná [Cauca Valley Montane Forests].

##### Notes

New record.

#### Wyeomyia (Nunezia) cf. paucartamboensis

Porter, 2014

59AA53E7-8BB4-5963-9C88-EE7356A5D2C6

##### Distribution

Caldas: Chinchiná [Cauca Valley Montane Forests].

##### Notes

New record.

#### Wyeomyia (Triamyia) aporonoma

Dyar & Knab, 1906

A37563ED-14AA-5557-A03D-0EE293638A79

##### Distribution

Caldas: Chinchiná [Cauca Valley Montane Forests]. Chocó: Nuquí [Chocó-Darién Moist Forests]. Valle del Cauca: Buenaventura [Chocó-Darién Moist Forests].

##### Notes

Reported by Barreto-Reyes (1955), Stone et al. (1959), Barreto and Vernon (1969), SIB (2020), new record.

#### Wyeomyia (Wyeomyia) cf. abebela

Dyar & Knab, 1908

81D9578B-919B-5EFC-A3AB-80911EA5ED34

##### Distribution

Caldas: Chinchiná [Cauca Valley Montane Forests]. Quindío: Quimbaya [Cauca Valley Montane Forests].

##### Notes

New records.

#### Wyeomyia (Wyeomyia) arthrostigma

(Lutz, 1905)

DA306BC3-19DE-58B6-98F9-0F27E759BC1F

##### Distribution

Cundinamarca: Soacha [Magdalena Valley Montane Forests]. Meta: Restrepo, Villavicencio [Apure-Villavicencio Dry Forests]. Valle del Cauca: Alcalá, Buenaventura, Cali[Cauca Valley Montane Forests, Chocó-Darién Moist Forests].

##### Notes

Reported by Heinemann and Belkin (1978), Carrejo and Gonzalez (1992), Suaza-Vasco et al. (2015), SIB (2020).

#### Wyeomyia (Wyeomyia) celaenocephala

Dyar & Knab, 1906

E27BBB4B-7E32-5A23-851A-BCBDBD5EF94A

##### Distribution

Antioquia: Apartadó, Jardín, Jericó [Magdalena Urabá Moist Forests, Cauca Valley Montane Forests]. Chocó: Litoral de San Juan, Nuquí [Chocó-Darién Moist Forests, South American Pacific Mangroves]. Valle del Cauca: Buenaventura [Chocó-Darién Moist Forests].

##### Notes

Reported by Barreto-Reyes (1955), Stone et al. (1959), Barreto and Vernon (1969), Parra-Henao and Suárez (2012), SIB (2020), new record.

#### Wyeomyia (Wyeomyia) cf. medioalbipes

Lutz, 1904

C4C35E72-5E04-563E-98D0-E099EF7A30D8

##### Distribution

Antioquia: Jericó [Cauca ValleyMontane Forests]. Caldas: Chinchiná [Cauca Valley Montane Forests]. Chocó: Acandí [Chocó-Darién Moist Forests]. Magdalena: Santa Marta [Guajira-Barranquilla Xeric Scrub].

##### Notes

New record.

#### Wyeomyia (Wyeomyia) melanopus

Dyar, 1919

C24F67FA-C27C-5DC6-9749-4112A27C9797

##### Distribution

Antioquia: Apartadó, Jericó, Turbo [Cauca Valley Montane Forests, Magdalena-Urabá Moist Forests]. Valle del Cauca: Buenaventura [Chocó-Darién Moist Forests].

##### Notes

Reported by Carrejo and Gonzalez (1992), Parra-Henao and Suárez (2012), SIB (2020), new record.

#### Wyeomyia (Wyeomyia) pertinans

(Williston, 1896)

30B4B557-BB09-5172-86DF-D7697E9745B9

##### Distribution

Chocó: Nuquí [Chocó-Darién Moist Forests]. Meta: Restrepo, Villavicencio [Apure-Villavicencio Dry Forests, Cordillera Oriental Montane Forests]. Valle del Cauca: Buenaventura, Dagua [Chocó-Darién Moist Forests, North-western Andean Montane Forests].

##### Notes

Reported by Heinemann and Belkin (1978), Carrejo and Gonzalez (1992), SIB (2020), new record.

#### Wyeomyia (Wyeomyia) scotinomus

(Dyar & Knab, 1907)

29E9A8C3-826D-569C-AF81-B207CD4AF447

##### Distribution

Atlántico: Barranquilla [Guajira-Barranquilla Xeric Scrub]. Antioquia: Fredonia, Hispania, Jericó [Cauca Valley Montane Forests]. Caldas: Chinchiná [Cauca Valley Montane Forests]. Chocó: Nuquí [Chocó-Darién Moist Forests]. Valle delCauca: Candelaria, Buenaventura. [Cauca Valley Dry Forests, Chocó-Darién Moist Forests].

##### Notes

Reported by Barreto-Reyes (1955), Stone et al. (1959), Barreto and Vernon (1969), new record.

#### Wyeomyia (Wyeomyia) simmsi

(Dyar & Knab, 1908)

C505D97D-3E92-5F76-9AE6-E7EA28D3CD5D

##### Distribution

Chocó: Nuquí [South American Pacific Mangroves]. Valle del Cauca: Buenaventura [Chocó-Darién Moist Forests].

##### Notes

Reported by Kano (1991), Carrejo and Gonzalez (1992), Zuluaga et al. (1993), SIB (2020).

## Analysis

**Figure 1.**
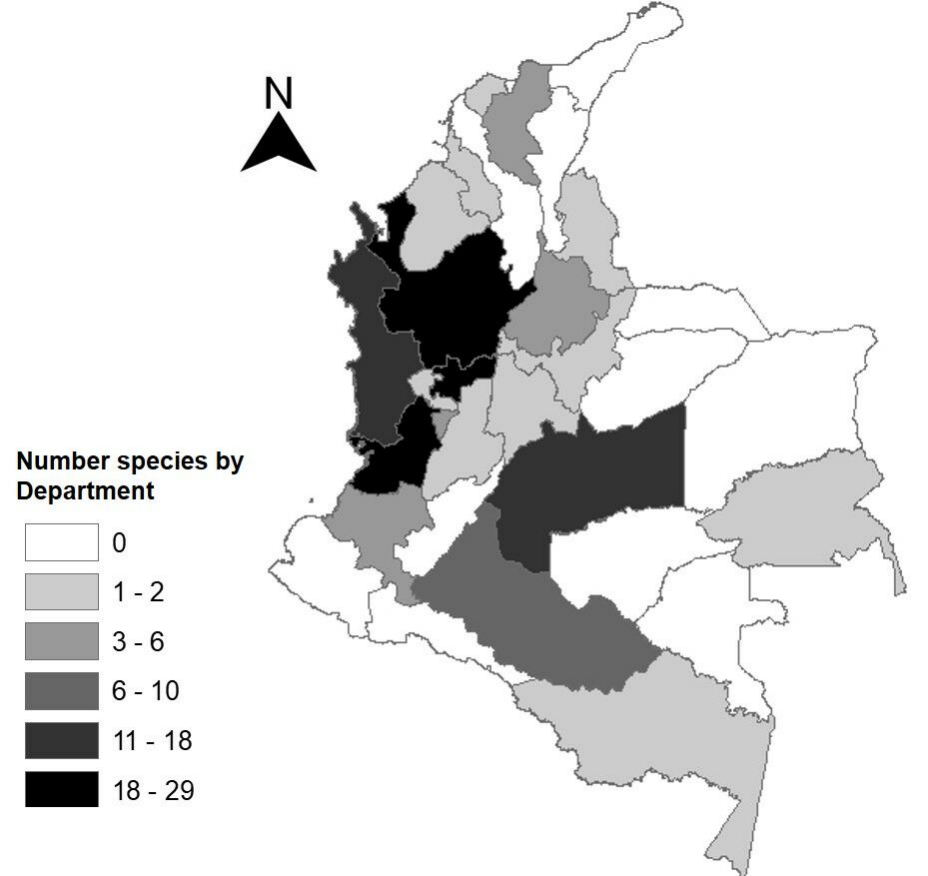
Political and administrative division of Colombia in Departments and distribution of the recorded species of tribe Sabethini. Darker areas indicate higher number of species (according to historical records available in literature and new records derived from this work).

This species checklist with distribution records of sabethine mosquitoes constitutes a first approximation to demonstrate the diversity of this group in Colombia. The results largely reflect the sampling efforts in specific ecoregions of the Colombian territory and the lack of studies in other areas with ecological conditions that may harbour species of Sabethini mosquitoes (Fig. 1).

A total of 68 species and 16 subgenera were recognised. *Wyeomyia* was the genus with the highest number of species (39). This genus exhibits the greatest number of species in the tribe (139) and, according to authorities, is prioritized for a taxonomic revision. Although *Wyeomyia* is divided into 17 subgenera, 29 species are without subgeneric placement (Harbach 2014, Pereira et al. 2019). Sabethini have been recorded in 19 of the 32 Departments of Colombia (Fig. 1) (Suppl. material 1). According to the literature review and the author's fieldwork, the genus *Trichoprosopon* exhibits the highest political distribution.

Colombia is also divided into 34 ecoregions (WWF 2015) and species listed here are present in 16 of them. The distribution records in ecoregions were obtained, based on the geographic coordinates (see Materials and Methods and Suppl. materials 1, 2). Chocó Darien Moist Forests, Cauca Valle Montane Forests and Apure-Villavicencio Dry Forests were the ecoregions with most distribution records of species: 38, 35 and 17 species, respectively. On the contrary, the Catatumbo Moist Forests, Negro-Branco Moist Forests, Amazon-Orinoco-Southern Caribbean Mangroves, North-western Andean Montane Forests and South American Pacific Mangroves were each represented by only a single genus.

Data indicate *Wyeomyia* occurs in 12 ecoregions. Chocó-Darién Moist Forests and Cauca Valley Montane Forests included the greatest number of species of this genus with a total of 26 and 19, respectively. Remarkably, *Wyeomyia* was the unique genus present in mangrove ecoregions. *Limatus* exhibit a wide distribution with presence in 10 ecoregions. This genus is represented by two species in the country, *L.asulleptus* and *L.durhamii*, the last one exhibiting the most cosmopolitan distribution in the tribe with presence in eight ecoregions.

Species of the genus *Sabethes* are involved in the transmission of very important arobovirus, such as yellow fever and Mayaro virus (Barrett and Higgs 2007, Muñoz and Navarro 2012, Navarro et al. 2015); however, species of *Trichoprosopon*, *Limatus* and *Wyeomyia* genera are important in the transmission of lesser-known viruses, such as Pixuna, Kairi, Ilheus, Guama and Caraparu (de Rodaniche and Galindo 1961, Karabatsos 1985, Navarro et al. 2015). Despite the growing importance in Colombia due to ecological and epidemiological changes, studies considering Sabethini distribution (Suaza-Vasco et al. 2015) and arbovirus transmission (Groot Liévano 2017) are limited.

In this study, the ecoregions with high numbers of Sabethini species contain known or suspected vector species. In Chocó Darien Moist Forests, an annual average of 16,000 mm precipitation (WWF 2015) may favour the existence of the phytotelmata used as breeding places for species, such as *J.ulopus* (Consoli and Oliveira 1994) and *L.assulleptus*, both of which are vectors of Mayaro virus (Navarro et al. 2015), *Sa.cyaneus* the vector of yellow fever, Mayaro virus and Ilhéus virus (Monath 1988, Navarro et al. 2015), Sa. *chloropterus* the vector of yellow fever, Ilhéus virus and St. Louis encephalitis virus (De Rodaniche and Galindo 1957, Monath 1988), *T.digitatum* the vector of St. Louis encephalitis virus, Bussuquara virus and Pixuna Hastrister et al. 1998, Auguste et al. 2010, as well as *T.pallidiventer*, a potential vector of Guama virus (De Souza Lopes et al. 1975, Navarro et al. 2015).

The Cauca Valley Montane Forests exhibit humid forest of the lower elevations (<1500 ma.s.l.) (WWF 2015) and have species, such as *J.ulopus* and *L.durhamii* known as the vectors of Venezuelan equine encephalitis and Caraparu virus (Aitken 1972, Navarro et al. 2015); *Sa.chloropterus* and *Sa.glaucodaemon* the vectors of yellow fever (Monath 1988); *T.compressum* the potential vector of Guama virus (Navarro et al. 2015), as well as *T.pallidiventer* and *T.digitatum*.

The Apure-Villavicencio Dry Forest is a transition zone between montane forests and extensive plains, composed of a mosaic of premontane forests, dry forests, savannah and gallery forests with low annual precipitation (WWF 2015). In this area are present potential vectors, such as *J.ulopus*, *L.assulleptus*, *L.durhamii*, *Sa.cyaneus*, *Sa.chloropterus*, *T.compressum* and *T.digitatum*. The Caquetá Moist Forest is part of Colombia Amazon with large expanses of seasonally-flooded forests (WWF 2015), where species of *Sabethes* with sylvatic preferences, such as *Sa.belisarioi* the vector of Ilhéus virus (Gravina et al. 2018), *Sa.cyaneus*, *Sa.chloropterus* and *Sa.glaucodaemon*, are present. *Limatusdurhamii* is also registered in this zone. The Magdalena Urabá Moist Forest is a region characterized by dry forests and wetland vegetation on flooded soils (WWF 2015) with data indicating the presence of species, such as *L.durhamii*; *Sa.cyaneus*; *T.compressum* and *T.digitatum*.

Two potential new species of the genus *Trichoprosopon* are mentioned in the distribution records (Suppl. material 1). They are named *Trichoprosopon* sp. n.e. A and *Trichoprosopon* sp. n.e. B. Both "species" are considered to be part of the *T.pallidiventer* complex (Suaza-Vasco et al. 2015). According to Zavortink (1981), some species of this complex can be sympatric. This was evidenced, based on revision of entomological material by the authors (not published data, including detailed morphological study of characters present in male genitalia, larvae and distribution records).

This work does not represent the complete distribution of the Sabethini tribe in Colombia, but it constitutes a first approximation to the more complete knowledge of the group in Colombia, including species and distribution. We consider ongoing studies to be relevant and intend to conduct a review of the material deposited in the entomological collections of museums and entities dedicated to the sampling of the Culicidae family for public health studies.

## Supplementary Material

XML Treatment for Isostomyia espini

XML Treatment for Johnbelkinia leucopus

XML Treatment for Johnbelkinia longipes

XML Treatment for Johnbelkinia ulopus

XML Treatment for Limatus asulleptus

XML Treatment for Limatus durhamii

XML Treatment for Onirion personatum

XML Treatment for Runchomyia (Ctenogoeldia) magna

XML Treatment for Sabethes (Peytonulus) identicus

XML Treatment for Sabethes (Peytonulus) ignotus

XML Treatment for Sabethes (Peytonulus) luxodens

XML Treatment for Sabethes (Peytonulus) undosus

XML Treatment for Sabethes (Peytonulus) xenismus

XML Treatment for Sabethes (Sabethes) albiprivus

XML Treatment for Sabethes (Sabethes) belisarioi

XML Treatment for Sabethes (Sabethes) cyaneus

XML Treatment for Sabethes (Sabethes) quasicyaneus

XML Treatment for Sabethes (Sabethes) tarsopus

XML Treatment for Sabethes (Sabethinus) intermedius

XML Treatment for Sabethes (Sabethinus) cf. xhyphydes

XML Treatment for Sabethes (Sabethoides) chloropterus

XML Treatment for Sabethes (Sabethoides) glaucodaemon

XML Treatment for Shannoniana fluviatilis

XML Treatment for Trichoprosopon andinum

XML Treatment for Trichoprosopon compressum

XML Treatment for Trichoprosopon digitatum

XML Treatment for Trichoprosopon evansae

XML Treatment for Trichoprosopon lanei

XML Treatment for Trichoprosopon pallidiventer

XML Treatment for Wyeomyia (subgenus uncertain) cf. argenteorostris

XML Treatment for Wyeomyia (subgenus uncertain) chalcocephala

XML Treatment for Wyeomyia (subgenus uncertain) clasoleuca

XML Treatment for Wyeomyia (subgenus uncertain) melanocephala

XML Treatment for Wyeomyia (subgenus uncertain) moerbista

XML Treatment for Wyeomyia (subgenus uncertain) phroso

XML Treatment for Wyeomyia (subgenus uncertain) serratoria

XML Treatment for Wyeomyia (subgenus uncertain) undulata

XML Treatment for Wyeomyia (Antunesmyia) colombiana

XML Treatment for Wyeomyia (Antunesmyia) flavifacies

XML Treatment for Wyeomyia (Cruzmyia) kummi

XML Treatment for Wyeomyia (Cruzmyia) mattinglyi

XML Treatment for Wyeomyia (Decamyia) cf. felicia

XML Treatment for Wyeomyia (Decamyia) pseudopecten

XML Treatment for Wyeomyia (Decamyia) ulocoma

XML Treatment for Wyeomyia (Dendromyia) complosa

XML Treatment for Wyeomyia (Dendromyia) jocosa

XML Treatment for Wyeomyia (Dendromyia) luteoventralis

XML Treatment for Wyeomyia (Dendromyia) ypsipola

XML Treatment for Wyeomyia (Dodecamyia) aphobema

XML Treatment for Wyeomyia (Exallomyia) tarsata

XML Treatment for Wyeomyia (Hystatomyia) chocoensis

XML Treatment for Wyeomyia (Hystatomyia) cf. circumcincta

XML Treatment for Wyeomyia (Hystatomyia) intonca

XML Treatment for Wyeomyia (Miamyia) codiocampa

XML Treatment for Wyeomyia (Miamyia) hosautos

XML Treatment for Wyeomyia (Miamyia) cf. limai

XML Treatment for Wyeomyia (Miamyia) oblita

XML Treatment for Wyeomyia (Nunezia) bicornis

XML Treatment for Wyeomyia (Nunezia) cf. paucartamboensis

XML Treatment for Wyeomyia (Triamyia) aporonoma

XML Treatment for Wyeomyia (Wyeomyia) cf. abebela

XML Treatment for Wyeomyia (Wyeomyia) arthrostigma

XML Treatment for Wyeomyia (Wyeomyia) celaenocephala

XML Treatment for Wyeomyia (Wyeomyia) cf. medioalbipes

XML Treatment for Wyeomyia (Wyeomyia) melanopus

XML Treatment for Wyeomyia (Wyeomyia) pertinans

XML Treatment for Wyeomyia (Wyeomyia) scotinomus

XML Treatment for Wyeomyia (Wyeomyia) simmsi

18FE2EEA-D81C-51F5-9350-68B55B02816D 10.3897/BDJ.10.e68413.suppl1 Supplementary material 1 New Colombian Sabethini records Data type Occurrences File: oo_539951.txt https://binary.pensoft.net/file/539951 Nelson Naranjo-Díaz

CD73B0C7-323A-58E1-8757-56D08B3CE0A4 10.3897/BDJ.10.e68413.suppl2 Supplementary material 2 References for records Data type References File: oo_539952.txt https://binary.pensoft.net/file/539952 Nelson Naranjo-Díaz

## References

[B6775272] Aitken T. H. (1972). Habits of some mosquito hosts of Venezuelan equine encephalitis (Mucambo) virus from northeastern South America, including Trinidad.

[B6775288] Auguste Albert J., Travassos da Rosa Amelia P. A., Adesiyun Abiodun A., Chadee Dave D., Tesh Robert B., Adams A. Paige, Arrigo Nicole C., Carrington Christine V. F., Martinez Raymond, Weaver Scott C. (2010). Isolation and characterization of sylvatic mosquito-borne viruses in Trinidad: Enzootic transmission and a new potential vector of Mucambo virus. The American Journal of Tropical Medicine and Hygiene.

[B6775312] Barajas J., Suaza J. D., Torres C., Leon Rua G., Uribe-Soto S., Porter C. H. (2013). Mosquitoes (Diptera: Culicidae) associated to guaduain municipalities of Anserma, Hispania and Jardin, Colombia. Revista Colombiana de Entomología.

[B6775323] Barrera Roberto, Liria Jonathan, Salas Rosalba, Boshell Jorge, Vasquez Clovis, Ahumada Marta, Weaver Scott C, Gonzalez Marta, Ferro Cristina, Freier Jerome, Navarro Juan-Carlos, Kang Wenli (2002). Contrasting sylvatic foci of Venezuelan equine encephalitis virus in northern South America.. The American Journal of Tropical Medicine and Hygiene.

[B6775355] Barreto P., Vernon L. (1969). Artrópodos hematófagos del río Raposo, Valle, Colombia: II - Culicidae. Caldasia.

[B6775420] Barreto-Reyes P. (1955). Lista de mosquitos de Colombia, S. A. (Diptera: Culicidae). Anales de la Sociedad de Biología.

[B6775429] Barrett Alan D. T., Higgs Stephen (2007). Yellow fever: A disease that has yet to be conquered. Annual Review of Entomology.

[B6775438] Bueno-Marí Rubén, Almeida A Paulo Gouveia, Navarro Juan Carlos (2015). Editorial: Emerging zoonoses: eco-epidemiology, involved mechanisms, and public health implications.. Frontiers in Public Health.

[B6775447] Carrejo N. S., Gonzalez R. (1992). Introducción al conocimiento de los Diptera.

[B6775455] Chaverri Luis Guillermo, Dillenbeck Claire, Lewis Devon, Rivera Cindy, Romero Luis Mario, Chaves Luis Fernando (2018). Mosquito species (Diptera: Culicidae) diversity from ovitraps in a Mesoamerican tropical rainforest. Journal of Medical Entomology.

[B6775515] Cochero Bustamante S. (2017). Identicación morfologica y molecular de especies del género *Haemagogus* (Diptera: Culicidae) en la región caribe colombiana.

[B6775588] Consoli R. A., Oliveira R. (1994). Principais mosquitos de importância sanitária no Brasil.

[B6775596] de Rodaniche Enid, Galindo Pedro (1961). Isolation of the virus of Ilhéus encephalitis from mosquitoes aptured in Panama. The American Journal of Tropical Medicine and Hygiene.

[B6775605] De Rodaniche Enid, Galindo Pedro (1957). Isolation of Ilhéus virus from *Sabetheschloropterus* captured in Guatemala in 1956. The American Journal of Tropical Medicine and Hygiene.

[B6775614] De Souza Lopes Oscar, Fonseca Ileana E. M., Lacerda José P. G., De Abreu Sacchetta Lia (1975). Bertioga (Guama Group) and Anhembi (Bunyamwera Group), two new arboviruses isolated in São Paulo, Brazil. The American Journal of Tropical Medicine and Hygiene.

[B6775623] Ferro María Cristina, Olano Victor Alberto, Ahumada Martha, Weaver Scott (2008). Mosquitos (Diptera: Culicidae) en el caserío de Chingalé, Santander, donde se registró un caso humano de encefalitis equina venezolana. Biomédica.

[B6775632] Gaffigan T. V., Wilkerson R. C., Pecor J. E., Stoffer J. A., Anderson T. Systematic catalog of Culicidae - Walter Reed biosystematics unit - home. http://www.mosquitocatalog.org.

[B6775641] Gravina Humberto Doriguêtto, Suzukawa Andreia Akemi, Zanluca Camila, Cardozo Segovia Fatima María, Tschá Marcel Kruchelski, Martins da Silva Allan, Faoro Helisson, da Silva Ribeiro Ricardo, Mendoza Torres Laura Patricia, Rojas Alejandra, Ferrerira Luis, Costa Ribeiro Magda Clara Vieira da, Delfraro Adriana, Duarte Dos Santos Claudia Nunes (2018). Identification of insect-specific flaviviruses in areas of Brazil and Paraguay experiencing endemic arbovirus transmission and the description of a novel flavivirus infecting *Sabethesbelisarioi*.. Virology.

[B6775660] Groot Liévano H. (2017). Estudios sobre virus transmitidos por artrópodos en Colombia. de la Academia Colombianade Ciencias Exactas, Físicas y Naturales.

[B6775669] Harbach Ralph E. (1994). The subgenusSabethinus of *Sabethes* (Diptera: Culicidae). Systematic Entomology.

[B6775678] Harbach Ralph E. (1995). Two new species of the subgenusPeytonulus of *Sabethes* (Diptera: Culicidae) from Colombia. Memórias do Instituto Oswaldo Cruz.

[B6775687] Harbach R., Peyton E. (2000). Systematics of *Onirion*, a new genus of Sabethini (Diptera: Culicidae) from the Neotropical region. Bulletin of the Natural History Museum.

[B6775696] Harbach R. E. Mosquito taxonomic inventory. http://mosquito-taxonomic-inventory.info/simpletaxonomy/term/6223.

[B6775718] Hastrister M. W., Lawyer P. G., Mauer D. J., Robbins R. G., Schultz G. W., Srtickman D. A. Disease vector ecology profile. www.afpmb.org/sites/%0Adefault/les/pubs/dveps/COLOMBIA.PDF.

[B6775728] Heinemann S., Belkin J. (1978). Collection records of the project ‘‘Mosquitoes of Middle America’’ 12 Colombia (COA, COB, COL,COM). Mosquito Systematics.

[B6775753] Kano T., Entomología Sociedad Colombiana de (1991). Inventario de mosquitos (Diptera: Culicidae) en algunas áreas del departamento del Chocó. XVIII Congreso de la Sociedad Colombiana de Entomología.

[B6775767] Karabatsos N. (1985). International catalogue of arboviruses including certain other virus of vertebrates.

[B6775775] Knight K. L., Stone A. (1977). Catalog of the mosquitoes of the world (Diptera: Culicidae).

[B6775783] Lane J., Cerqueira N. L. (1942). Os sabetíneos da América (Diptera, Culicidae).

[B6775791] Lane J. (1953). Neotropical Culicidae.

[B6775799] Marchon-Silva Verônica, Lourenço-de-Oliveira Ricardo, Almeida Magaly Dolsan de, Silva-Vasconcelos Adenildo da, Costa Jane (1996). The type specimens of mosquitoes (Diptera, Culicidae) deposited in the entomological collection of the Instituto Oswaldo Cruz, Rio de Janeiro, Brazil. Memórias do Instituto Oswaldo Cruz.

[B6775809] Molina Jorge A., Hildebrand Patricio, Olano Víctor A., Muñoz de Hoyos Paulina, Barreto Mauricio, Guhl Felipe (2000). Fauna de insectos hematófagos del sur del Parque Natural Nacional Chiribiquete, Caquetá, Colombia. Biomédica.

[B6775833] Monath T. P. (1988). The arboviruses: Epidemiology and ecology.

[B6775841] Muñoz Manuel, Navarro Juan Carlos (2012). Virus Mayaro: un arbovirus reemergente en Venezuela y Latinoamérica. Biomédica.

[B6775850] Navarro J. C., Arrivillaga J., Morales D., Ponce P., Cevallos V. (2015). Evaluación rápida de biodiversidad de mosquitos (Diptera: Culicidae) y riesgo en salud ambiental en un área Montana del Chocó Ecuatoriano. Entomotrópica.

[B6775860] Olano Víctor Alberto, Tinke Milton E. (1993). Fauna de mosquitos asociada con *Aedesaegypti* en Guaduas, Colombia. Biomédica.

[B6775869] Parra-Henao Gabriel, Suárez Laura (2012). Mosquitos (Díptera: Culicidae) vectores potenciales de arbovirus en la región de Urabá, noroccidente de Colombia. Biomédica.

[B6775878] Pereira Aldenice N., Talaga Stanislas, Guimarães Anthony Érico, Lourenço-De-oliveira Ricardo, Motta Monique De Albuquerque (2019). Taxonomic history of species without subgeneric placement in the genus *Wyeomyia* Theobald (Diptera: Culicidae) and recognition of *Wy.compta* Senevet & Abonnenc as a junior synonym of *Wy.argenteorostris* (Bonne-Wepster & Bonne). Zootaxa.

[B6775888] Porter Charles H., Wolff E. Marta I. (2004). A new species of Wyeomyia (Hystatomyia) (Diptera: Culicidae) from Colombia and a redescription of Wy. (Hystatomyia) intonca Dyar & Knab.. Zootaxa.

[B6775897] Rosero-García Doris, Bickersmith Sara A., Suaza-Vasco Juan David, Porter Charles, Correa Margarita M., Conn Jan E., Uribe-Soto Sandra (2017). Molecular operational taxonomic units of mosquitoes (Diptera: Culicidae) collected in high Andean mountain ecosystems of Antioquia, Colombia. Zootaxa.

[B6775909] Rosero-García Doris, Rúa-Uribe Guillermo, Correa Margarita M, Conn Jan E, Uribe-Soto Sandra (2018). Mosquito (Diptera: Culicidae) grouping based on larval habitat characteristics in high mountain ecosystems of Antioquia, Colombia.. Journal of Vector Ecology.

[B6775919] Rozo-Lopez Paula, Mengual Ximo (2015). Updated list of the mosquitoes of Colombia (Diptera: Culicidae).. Biodiversity Data Journal.

[B6775936] Shope R. E., Woodall J. P., da Rosa A. T. (2000). The Arboviruses: Epidemiology and Ecology.

[B6775949] SIB Sistema de información sobre diversidad de Colombia. https://sibcolombia.net.

[B6775957] Sigovini M., Keppel E., Taliapetra D. (2016). Open nomenclature in the biodiversity era. Methods in Ecology and Evolution.

[B6775979] Stone A., Knight K. L., Starcke H. (1959). Synoptic catalog of the mosquitoes of the world (Diptera, Culicidae).

[B6775987] Suaza-Vasco Juan, López-Rubio Andrés, Galeano Juan, Uribe Sandra, Vélez Iván, Porter Charles (2015). The Sabethines of northern Andean coffee-growing regions of Colombia. Journal of the American Mosquito Control Association.

[B6775998] Vargas L., Díaz Nájera A. (1959). Descripción del macho de Sabethes (Sabethes) belisarioi Neiva, 1908. Nueva especie para México (Insecta: Diptera). Revista del Instituto de Salubridad y Enfermedades Tropicales.

[B6776060] Worth C B, Downs W G, Aitken T H, Tikasingh E S (1968). Arbovirus studies in Bush Bush Forest, Trinidad, W. I., September 1959 - December 1964. IV. Vertebrate populations.. The American Journal of Tropical Medicine and Hygiene.

[B6776007] WWF List of ecoregions. http://wwf.panda.org/about_our_earth/ecoregions/ecoregion_list/.

[B6776024] Zavortink T. (1979). Mosquito studies (Diptera, Culicidae) XXXV. The new sabethine genus *Johnbelkinia* and a preliminary reclassification of the composite genus *Trichoprosopon*.

[B6776032] Zavortink T. (1979). A reclassification of the sabethine genus *Trichoprosopon*. Mosquito Systematics.

[B6776041] Zavortink T. (1981). Species complexes in the genus *Trichoprosopon*. Mosquito Systematics.

[B6776050] Zuluaga J., Zuluaga J. S., Weiser J., Rojas W., Orduz S. (1993). Microsporidia parásitos de larvas de mosquito de la Costa Pacífica del Chocó. Caldasia.

